# Digital Financial Literacy and Life Satisfaction: Evidence from South Korea

**DOI:** 10.3390/bs15010094

**Published:** 2025-01-20

**Authors:** Youngjoo Choung, Tae-Young Pak, Swarn Chatterjee

**Affiliations:** 1 Department of Consumer Science, Inha University, Incheon 22212, Republic of Korea; joo@inha.ac.kr; 2 Department of Consumer Science and Convergence Program for Social Innovation, Sungkyunkwan University, Seoul 03063, Republic of Korea; typak@skku.edu; 3 Department of Financial Planning, Housing and Consumer Economics, University of Georgia, Athens, GA 30602, USA

**Keywords:** digital financial literacy, digital financial capability, digital financial inclusion, financial literacy, life satisfaction

## Abstract

Digital financial literacy is an emerging concept that refers to the ability to effectively use digital tools, platforms, and services to manage personal finances. While previous studies have explored the behavioral effects of digital financial literacy, less is known about its broader well-being implications for financial consumers. In this study, we aim to examine the association between digital financial literacy and life satisfaction in a developed country context. Digital financial literacy was measured using a multidimensional scale that encompasses financial knowledge, digital literacy, digital financial service awareness, practical know-how of digital financial services, and self-protection against digital financial fraud. Using data of 1615 Korean adults who currently use digital financial services, this study estimated a series of regressions linking life satisfaction to digital financial literacy and covariates. The results showed a significant association between digital financial literacy and life satisfaction, with self-protection against fraud being the most influential subdimension. Notably, financial knowledge was not associated with life satisfaction when other dimensions of digital financial literacy were independently included in the regression models. These findings highlight the importance of digital skills and knowledge in navigating digital financial services. They also emphasize the need for targeted policies, financial education initiatives, and consumer protection measures to address the lack of digital financial literacy among marginalized populations.

## 1. Introduction

Financial products and services have become increasingly digitalized and accessible through online platforms and applications. Since the onset of the COVID-19 pandemic, the adoption of digital financial services and contactless transactions has accelerated globally, driving the rapid digital transformation of the financial services industry (Raj et al., 2023). Digital financial services now encompass a wide range of offerings, including savings, investments, insurance, and loans, as well as routine transactions like payments and remittances. This shift highlights the growing integration of digital technologies into the financial sector, reshaping how financial services are delivered and accessed (Hu & Zheng, 2016).

Digital financial services enable consumers to access financial products and services more conveniently. However, the widespread adoption of digital financial services has introduced new challenges. One notable issue is the digital divide, which disproportionately affects socially vulnerable groups such as the elderly, low-income individuals, and people with disabilities (Zheng & Wang, 2024). In South Korea, there is growing concern that many older adults may lack the digital skills required to access and use digital financial services. For older adults, digital exclusion is a major social issue that could restrict access to financial services and undermines their overall well-being (Zheng & Wang, 2024).

The rapid expansion of digital financial services also raises concerns about the safety of digital financial products and services. These risks include cybersecurity threats such as online fraud, data breaches, and hacking, as well as potential financial instability stemming from the excessive use of consumer credit (Liu & Hou, 2023). These multifaceted risks pose challenges for consumer protection and the stability of financial systems. Addressing these issues requires a deeper understanding of digital finance, coupled with comprehensive regulatory measures and education initiatives to ensure its safe and equitable use (Eichengreen, 2023).

The digital financial landscape requires consumers to develop a comprehensive understanding of the risks and opportunities in digital finance, as well as the ability to navigate these services effectively. In this context, digital financial literacy (DFL) has emerged as a vital competency for financial consumers in the digital era. DFL is a multidimensional concept that integrates “knowledge, skills, confidence and competencies to safely use digitally delivered financial products and services” (Alliance for Financial Inclusion, 2021). It goes beyond traditional financial literacy by including the understanding and effective use of modern financial services on digital platforms (OECD, 2018). The OECD/INFE recently incorporated a DFL assessment tool into its International Survey of Adult Financial Literacy, allowing the calculation of the DFL score (OECD, 2024).

The emerging literature has explored the conceptualization and measurement of DFL across various contexts (Golden & Cordie, 2022; Koskelainen et al., 2023; Lyons & Kass-Hanna, 2021; Morgan et al., 2019; Ravikumar et al., 2022). Studies like those of Lyons and Kass-Hanna (2021) and Ravikumar et al. (2022) have proposed a multidimensional framework for DFL, which collectively assesses knowledge, skills, attitudes, and behaviors related to the access and use of digital financial services. Empirical research has found that higher levels of DFL are associated with increased use of digital financial services, including online banking, mobile payments, and digital investments (Shehadeh et al., 2024; Tony & Desai, 2020; Widyastuti et al., 2024). Some studies suggest that DFL affects saving, investment, and spending behaviors by empowering individuals to effectively utilize digital platforms for financial decision making and management (Rahayu et al., 2022a; Setiawan et al., 2022; Yadav & Banerji, 2024).

To date, most of the existing studies have focused on developing countries and have primarily explored specific financial behaviors or outcomes to assess the effects of DFL. Furthermore, there has been limited research on whether DFL contributes to broader life satisfaction beyond personal finances. Given that life satisfaction serves as the ultimate measure of well-being (Diener et al., 1985), the objective of this study is to examine the potential association between DFL and life satisfaction. We explore this issue in the context of South Korea—a country with advanced digital infrastructure and the widespread adoption of digital financial services. Unlike in many developing countries, where financial inclusion remains a significant challenge, South Korea presents a unique opportunity to study how variations in DFL within a digitally integrated society influence life satisfaction. This study setting broadens our understanding of DFL’s role in fostering financial inclusion, as well as in enhancing subjective well-being among individual consumers.

DFL can influence life satisfaction through multiple mechanisms, both positive and negative. Individuals with higher levels of DFL are likely to use digital financial services more effectively and experience greater financial security and a stronger sense of control over their personal finances (Ozili, 2018). Given that financial well-being is an important dimension of life, this channel could boost life satisfaction. However, DFL can also have a negative association with life satisfaction, particularly when increased access to digital financial services leads to unintended consequences. For instance, while greater use of digital financial platforms provides convenience, it also exposes users to risks such as online fraud, identity theft, and data breaches (Hasham et al., 2019). Additionally, individuals with high DFL might engage in excessive risk-taking behaviors, as digital finance could promote stock market participation and over-leveraging through mobile platforms (Lu et al., 2024; Wang et al., 2023; Ye et al., 2022). The complexity of digital financial tools can also create a sense of anxiety for some individuals, potentially diminishing the overall benefits of digital finance (H. N. Kim et al., 2023). Thus, while DFL has the potential to enhance life satisfaction, its impact is contingent on the individual’s ability to navigate the risks associated with digital finance.

## 2. Literature Review

### 2.1. Financial Literacy, Financial Behavior, and Life Satisfaction

Prior to the emergence of digital finance, financial literacy was widely recognized as a key predictor of individuals’ financial competencies, and numerous studies have been conducted on this topic both domestically and internationally (Huston, 2010; Lusardi & Mitchell, 2007, 2009; Pak, 2018). Lusardi and Mitchell (2007, 2009) measured individuals’ basic financial knowledge using items on concepts such as compound interest, inflation, and risk diversification. These items have become the global standard for assessing financial literacy. The OECD/INFE measures financial literacy through financial knowledge, attitudes, and behaviors, and similar surveys are conducted in South Korea (Bank of Korea & Financial Supervisory Service, 2023).

Research on financial literacy has explored a variety of perspectives. Early studies in South Korea primarily emphasized the importance of financial education across different age groups (Choe & Cho, 2011; M. J. Kim et al., 2012; Y. H. Lee, 2015, 2018; Pak et al., 2024). Additionally, many studies have investigated the relationship between financial literacy, asset accumulation, and financial behavior. Van Rooij et al. (2011) found that individuals with low financial literacy were less prepared for retirement and asset accumulation. Lusardi and Mitchell (2007) highlighted the positive role of financial literacy in long-term asset accumulation and retirement planning among baby boomers. Similar findings have been reported in South Korea, where financially literate consumers were found to hold diverse financial assets (Jeong & Kim, 2016), and those with low financial literacy showed lower demand for private pensions (Son & Park, 2022). Kaiser and Menkhoff (2017) explored the link between financial literacy and financial behavior and found that individuals with higher financial literacy were better prepared for retirement and accumulated more assets. Similarly, Pak and Chatterjee (2016) revealed that households with high financial literacy but low financial practice skills were less likely to maintain precautionary savings, possibly due to overconfidence and an illusion of control over uncertain financial outcomes.

The previous literature has also examined the relationship between financial literacy and life satisfaction. The findings suggest that higher financial literacy contributes to increased financial satisfaction through effective asset management and financial planning (Iqbal et al., 2023; Zhang & Chatterjee, 2023). Lusardi and Mitchell (2011) emphasized that individuals with high levels of financial literacy are more likely to make wise financial plans and succeed, significantly impacting their lifelong well-being. Mitra and De (2024), in their study of rural households in India, found that financial literacy directly and indirectly influences life satisfaction. Choi (2023) highlighted that financial competencies in the technological domain positively affect both financial satisfaction and overall life satisfaction.

### 2.2. Digital Financial Literacy, Financial Behavior, and Life Satisfaction

While the concept of financial literacy is primarily focused on understanding personal finance principles, DFL encompasses broader aspects of how individuals access and use financial services offered through digital platforms. Early definitions conceptualized DFL as the ability to use digital tools to make informed financial decisions while protecting oneself from digital financial risks, emphasizing the ability to avoid security threats (Morgan et al., 2019). More recent studies have expanded the definition to include the knowledge, skills, attitudes, and behaviors that are necessary to safely and effectively use digital financial services to enhance financial well-being (Alliance for Financial Inclusion, 2021; Koskelainen et al., 2023; Lyons & Kass-Hanna, 2021; Ravikumar et al., 2022). DFL is considered a specific domain of financial capability involving digital finance (OECD, 2024). The traditional concept of financial literacy overlooks skills and risks specific to the digital environment and is thus unable to fully capture how individuals make decisions and engage with digital financial services (Lyons & Kass-Hanna, 2021).

Findings from the previous literature suggest that DFL is significantly associated with financial behaviors and the use of digital financial services. Morgan and Long (2019) found a significant association between financial literacy and a positive perception of fintech products. Similarly, Yang et al. (2020) attributed low digital financial usage rates in China to a lack of financial literacy. DFL has been shown to predict current and future financial behaviors involving long-term financial planning (Abdallah et al., 2024; Setiawan et al., 2022). It was found to promote the adoption of cashless payments and mobile banking and bridge gaps across gender and regions (Shehadeh et al., 2024; Phan et al., 2024). Studies have also revealed the associations between DFL, demographic factors, and digital financial inclusion (Normawati et al., 2022; Widyastuti et al., 2024). A study by Umar and Dalimunthe (2024) found that financial literacy and digital literacy are indirectly associated with the recognition of investment fraud, and this association is mediated by cybercrime awareness.

Several studies have identified DFL as a significant predictor of financial well-being. Choung et al. (2023) found that DFL is positively associated with financial well-being, and this association is mainly due to the ability to self-protect from digital fraud. Similarly, Rahayu et al. (2022b) reported that DFL is associated with reductions in financial problems and higher financial well-being in Indonesia. Studies like those by Jhonson et al. (2023) and Respati et al. (2023) suggest that DFL could influence financial well-being by improving saving, spending, and investment behaviors. The research further emphasizes the role of DFL in fostering informed financial decisions and overall financial well-being (Kamble et al., 2024; Kumar et al., 2023). However, the existing studies are focused on the relationship between DFL and financial well-being, and not much is known about whether this financial well-being benefit translates into higher life satisfaction. In particular, studies focusing on Korean financial consumers are scarce, and this underscores the need for research evaluating DFL among Korean consumers and its impact on life satisfaction.

A handful of studies have shown that the association between DFL and financial behaviors or well-being varies across sociodemographic groups. For instance, Yang et al. (2020) demonstrated that the impact of financial literacy on digital finance adoption in China is more pronounced among wealthy, high-income, younger households, women, and urban residents. Similarly, Azeez and Akhtar (2021) emphasized that DFL levels differ significantly based on sociodemographic factors, including education, income, and gender. Prasad et al. (2018) revealed that disparities in DFL across income and education levels influence digital finance adoption in Udaipur, India. Finally, Choung et al. (2023) examined DFL among Korean adults and found that the association between self-protection abilities and financial well-being remains significant across various sociodemographic subgroups. So far, the evidence remains mixed, and further research is needed to identify which subgroups are more vulnerable to a lack of DFL.

### 2.3. Research Questions

This study aims to explore the potential association of DFL with life satisfaction among Korean adults. By considering the related literature and the multidimensional nature of DFL, the following research questions were formulated:

1. How is DFL related to life satisfaction?
2. How are the subdimensions of DFL related to life satisfaction?

Testing these research questions would deepen our understanding of the well-being effects of DFL by moving beyond traditional financial outcomes to assess its broader implications for subjective well-being. By analyzing both the overall impact of DFL and the specific contributions of its subdimensions, this study provides nuanced insights into which aspects of DFL are most critical for enhancing life satisfaction.

## 3. Method

### 3.1. Data Collection

The sample for this study was drawn from the panel database of Macromill Embrain, which includes more than one million registered panel members. Email invitations containing the survey link were sent to individuals who met the inclusion criteria (ages 25 to 59, currently using any digital financial service), and a total of 1615 respondents were selected. Stratified sampling was used to ensure that the sample was representative of the Korean population in terms of age, gender, and region. The survey was conducted online from April to May 2023, and participants were briefed that they would be answering questions about financial capability and well-being. Per IRB approval for this study (SKKU 2023-04-046), participants signed an informed consent form and were notified that their responses would be anonymized and used exclusively for research purposes. Respondents who completed the survey received compensation from the research firm at a rate of KRW 1000 per minute. The sample characteristics are summarized in Table 1.

### 3.2. Digital Financial Literacy

This study used a multidimensional scale of DFL by Lyons and Kass-Hanna (2021), adapted to the Korean context. This scale encompasses five dimensions—financial knowledge, digital literacy, digital financial service awareness, practical know-how of digital financial services, and self-protection against digital financial fraud—that are directly related to the competencies required for the appropriate use of digital financial services (see Table A1 for the measurement items).

Financial knowledge was evaluated using the OECD/INFE International Survey of Adult Financial Literacy (OECD, 2018). This assessment includes seven items covering topics such as the time value of money, interest on loans, simple and compound interest, risk and return, inflation, and risk diversification. Each correct answer is awarded one point, resulting in a financial knowledge score ranging from 0 to 7.

Digital literacy consists of 10 items that assess basic knowledge of digital hardware and software use. These items measure understanding of basic digital device operations, authentication methods, internet connectivity, and application management.

Digital financial service awareness is assessed through eight items that examine knowledge of the available digital solutions for online and mobile financial transactions. These items evaluate familiarity with various services, including internet banking, mobile investments, online loans, and digital payment systems.

Practical know-how of digital financial services is assessed through seven items that measure the ability to effectively use software and mobile applications for managing personal finances. These items evaluate essential technical skills, including signing up for digital financial services, completing self-authentication processes, performing basic financial transactions, utilizing simple payment methods, and resolving errors.

Self-protection against digital financial fraud is assessed through eight items that measure the ability to detect and prevent fraudulent activities on digital platforms. These items evaluate awareness and response capabilities for various digital financial fraud risks, including avoiding unnecessary fees, identifying bait products, managing passwords, preventing phishing, dealing with malware, and recognizing voice phishing.

Participants rated their responses on a 5-point Likert scale (1 = strongly disagree to 5 = strongly agree), and the average scores were computed for each subdimension. An aggregate DFL index was calculated by summing the scores of the five subdimensions, resulting in a range of 4 to 27. To simplify the interpretation of the coefficient estimates, the comprehensive index and subdimension scores were standardized using z-score normalization.

### 3.3. Life Satisfaction

Participants were presented with a visual analogue of a thermometer, with 0 indicating the worst possible life and 100 indicating the best possible life. They were then asked to think about their life as a whole and determine where their level of life satisfaction falls on this scale. This “feeling thermometer” approach has been widely used in the well-being literature to measure various satisfaction or distress measures on a continuum of 0 to 100 (Choung et al., 2021; Harju et al., 2019; S. J. Lee & Wu, 2008; Mellor et al., 1999). As a single-item measure, it is easy to understand and helps to avoid cultural biases and complications that can arise when interpreting multi-item scales across diverse populations (Converse & Presser, 1986). Research has validated the single-item life satisfaction measures (Cheung & Lucas, 2014; Lucas & Donnellan, 2012) and the thermometer scale of life satisfaction in comparison to other rating scales (Alwin, 1997).

### 3.4. Analyses

Linear regression was employed to estimate the relationship between DFL and life satisfaction, as it is suitable for a continuous dependent variable (Fox, 2015). Our regression models were adjusted for demographic and socioeconomic controls, including age, gender, education background, marital status, number of household members, self-rated health, household income, financial assets, poverty status, housing type, and province dummies. We began with estimating a correlation between a DFL score and life satisfaction and gradually augmenting regressions with demographic and socioeconomic variables using a hierarchical linear regression approach (Field, 2024). We then estimated the association between each dimension of DFL and life satisfaction to identify the subdimensions that significantly influence life satisfaction. Our regression method allows for a step-by-step evaluation of the hypothesized relationship and for testing its heterogeneity based on the subdimensions. All analyses were performed using Stata MP 16.1 (StataCorp, College Station, TX, USA). Statistical significance was determined at a *p*-value threshold of 0.05.

## 4. Results

The summary statistics provide an overview of the sample characteristics across the variables (Table 1). The full sample comprised 1615 respondents with an average life satisfaction score of 60.92 (SD = 19.17). The mean DFL score was 22.65 (SD = 2.88), and the participants demonstrated varying competencies across the five subdimensions of DFL: financial knowledge (mean = 5.44, SD = 1.45), digital literacy (mean = 4.62, SD = 0.56), digital financial service awareness (mean = 4.43, SD = 0.62), practical know-how of digital financial services (mean = 4.40, SD = 0.61), and self-protection against digital financial fraud (mean = 3.75, SD = 0.65). The average age of the participants was 42.63 years (SD = 9.67), and the majority reported good self-rated health (88.5%), with a household size of approximately three members on average (mean = 2.93, SD = 1.22). Homeownership was reported by 60.1% of the participants, while the rest lived in long-term rentals (17.9%) or other arrangements (22%).

When disaggregated by gender, men and women showed slight variations in life satisfaction, DFL, and its subdimensions. Men reported an average life satisfaction score of 59.83, compared to 62.06 for women. Across all the subdimensions of DFL, men generally reported higher scores in financial knowledge (5.58 vs. 5.30) and self-protection against digital financial fraud (3.81 vs. 3.70). Both genders had similar scores in digital literacy (4.62 vs. 4.61) and practical know-how of digital financial services (4.40 for both). The average age of the sample was 42.63 years, and about 51.2% were men.

When categorized by DFL levels (above or below the median), respondents with higher DFL scores reported greater life satisfaction (63.22 vs. 58.61) and stronger performances across all the subdimensions of DFL. For example, the high-DFL group scored significantly higher in financial knowledge (6.24 vs. 4.64), self-protection against digital financial fraud (4.08 vs. 3.43), and digital financial service awareness (4.81 vs. 4.06). Demographically, the high-DFL group was slightly younger on average (41.34 years vs. 43.91 years) and included a larger proportion of individuals with postgraduate degrees (15.7% vs. 7.3%). Additionally, this group had higher average financial assets and household income. These patterns suggest that higher DFL is associated with both improved life satisfaction and stronger socioeconomic standing.

The correlation matrix shows positive relationships between life satisfaction, DFL, and its subdimensions (Table 2). Life satisfaction is modestly correlated with DFL (r = 0.149), self-protection against digital financial fraud (r = 0.138), awareness of digital financial services (r = 0.132), practical know-how of digital financial services (r = 0.125), and digital literacy (r = 0.115). When restricted to correlations between the subdimensions, the strongest correlations are observed for awareness of digital financial services and practical know-how (r = 0.839), followed by digital literacy and awareness of digital financial services (r = 0.735). Financial knowledge has weaker correlations with life satisfaction (r = 0.083) and the other subdimensions.

The results of the regressions regarding the association of DFL with life satisfaction are presented in Table 3. Across all three regression models, DFL demonstrated a statistically significant association with life satisfaction at the 5% level. In the baseline model (column 1), a one-standard-deviation increase in DFL was associated with a 2.86-point increase in life satisfaction, or about a 4.7% increase from the sample mean of 60.92. The inclusion of demographic controls in the second model (column 2) slightly reduced the coefficient estimate, with a one-standard-deviation increase in DFL resulting in a 1.95-point increase in life satisfaction, or about a 3.2% increase from the sample mean. In the full model (column 3), which controls for socioeconomic variables and province fixed effects, a one-standard-deviation increase in DFL led to a 1.752-point or 2.9% increase in life satisfaction. Across all three models, the association between DFL and life satisfaction was robust to accounting for demographic and socioeconomic factors. Among the control variables, self-rated health had the strongest association with life satisfaction, with individuals rating their health as average or better, reporting 18.11-point-higher life satisfaction. Being married and having a postgraduate degree also exhibited significant associations with life satisfaction, contributing to increases of 6.73 and 11.23 points, respectively. However, living in monthly rental housing was associated with a significant 4.69-point reduction in life satisfaction compared to homeowners.

The regressions in Table 4 explore the associations between the DFL subdimensions and life satisfaction. In the first five columns, each subdimension of DFL—financial knowledge, digital literacy, awareness of digital financial services, practical know-how of DFS, and self-protection against digital financial fraud—is examined separately for its association with life satisfaction. Financial knowledge is positively associated with life satisfaction, but it is not significant at the 5% level (β = 0.053, *p* = 0.85). Digital literacy (β = 1.606, *p* < 0.01), awareness of digital financial services (β = 1.313, *p* < 0.01), practical know-how of digital financial services (β = 1.432, *p* < 0.01), and self-protection against digital financial fraud (β = 1.687, *p* < 0.01) show significant positive associations with life satisfaction.

When all the subdimensions are included simultaneously (column 6), the significance of most of the subdimensions substantially diminishes, except for self-protection against digital financial fraud (β = 1.318, *p* = 0.013), which remains significant at the 5% level. This reduction in significance suggests potential multicollinearity among the subdimensions, where overlapping contributions reduce the independent explanatory power of each subdimension. The persistence of the self-protection score as a significant predictor highlights its robust association with life satisfaction, even after accounting for other dimensions of DFL.

In the full model with the DFL score (column 7), a one-unit increase in overall DFL is associated with a 1.752-point increase in life satisfaction (*p* < 0.01), or about a 2.9% increase from the sample mean. The adjusted R^2^ is about 0.22–0.23 across all regressions. Overall, these regression results show that DFL is a significant predictor of life satisfaction, and that this association is mostly driven by the significant influence of self-protection against digital financial fraud.

## 5. Discussion

This study highlights the growing importance of digital financial literacy (DFL) in the evolving digital financial landscape. By examining the relationship between DFL and life satisfaction, it offers empirical evidence on how digital financial competencies influence overall well-being. DFL is conceptualized as a multidimensional construct that encompasses financial knowledge, digital literacy, awareness of digital financial services, practical skills, and self-protection against digital financial fraud (Lyons & Kass-Hanna, 2021; OECD, 2024). This comprehensive approach considers the knowledge, attitude, behavior, and skills related to digital finance to provide a holistic evaluation of how digital financial capabilities affect well-being in the digital age.

The results revealed that DFL is positively associated with life satisfaction, and the association remained significant after accounting for demographic and socioeconomic factors. Among the subdimensions of DFL, self-protection against digital financial fraud was identified as the most influential predictor of life satisfaction. Notably, financial knowledge was not associated with life satisfaction when other dimensions of DFL were controlled for in the regression. These findings indicate that basic financial knowledge alone is insufficient to capture the overall well-being effects of financial capability in the digital financial landscape. They also highlight that, among Korean adults, the ability to protect against digital fraud plays a more critical role in enhancing life satisfaction than basic financial knowledge.

The association between DFL and life satisfaction aligns with previous research highlighting the role of DFL in improving financial well-being (Choung et al., 2023; Jhonson et al., 2023; Kamble et al., 2024; Kumar et al., 2023; Rahayu et al., 2022b; Respati et al., 2023). While this study could not explore the potential mechanisms underlying this association, previous studies have shown that DFL enhances decision making regarding saving, spending, and investing through digital platforms, and that these combined effects contribute to higher financial well-being (Jhonson et al., 2023; Respati et al., 2023). Our findings contribute to the literature by demonstrating that the well-being effects of DFL may extend beyond financial well-being to improve overall life satisfaction, which encompasses both financial and non-financial aspects of well-being.

Our finding that the self-protection ability predicts life satisfaction corroborates Morgan et al. (2019)’s definition of DFL, which emphasizes the ability to avoid digital security threats. This finding is particularly relevant to South Korea, which has experienced a surge in digital financial crimes (Korean National Police Agency, 2023). A report from the Korean Financial Supervisory Service indicates that digital phishing scams, including voice phishing and fraudulent text messages, have caused significant financial losses among vulnerable populations, including the elderly and those less familiar with digital financial systems (Korean Financial Supervisory Service, 2024). The ability to self-protect against such threats is therefore not only a critical component of DFL but also a vital factor in safeguarding individuals’ overall well-being. By equipping individuals with the skills to recognize and avoid these threats, DFL directly contributes to reducing the psychological and financial burdens associated with digital financial fraud. Our findings suggest that enhancing the self-protection ability as part of DFL education could serve as an effective strategy for mitigating the impact of digital financial crimes in South Korea and improving both the financial and life satisfaction of Korean financial consumers. Governments and financial authorities should also consider targeted policies to improve DFL across diverse demographic groups and further expand existing financial protection laws to explicitly cover digital domains.

A notable limitation of this study is the potential lack of generalizability due to sampling bias. The data were collected from an online panel of adults in South Korea, which may not fully represent the broader population. While stratified sampling was used to ensure representation across age, gender, and region, using an online survey platform may have excluded individuals with limited internet access or those less familiar with digital technologies. Additionally, the use of a single-item scale to measure life satisfaction may not fully capture its multidimensional nature. Although the study controls for key demographic and socioeconomic variables, unobserved factors such as psychological well-being and social relationships could contribute to unexplained variance.

Future research should consider longitudinal designs to better understand the long-term impacts of DFL on life satisfaction. Such studies can provide valuable insights into how digital financial competencies evolve over time and their sustained effects on various aspects of well-being. Additionally, exploring causal pathways between DFL and life satisfaction using instrumental variables or natural experiments would deepen our understanding of this relationship. Comparative studies across diverse cultural and economic contexts could also shed light on how digital financial environments influence life satisfaction in different countries. Addressing these gaps would contribute to more effective policies and educational interventions aimed at promoting digital financial inclusion and overall well-being.

In conclusion, this study demonstrates the significant role of DFL in enhancing life satisfaction, with self-protection against digital financial fraud identified as the most impactful factor. The findings emphasize the need for robust policies and targeted educational initiatives to empower individuals with the skills and knowledge to navigate digital finance safely. By demonstrating the broader well-being implications of DFL, this study underscores the importance of aligning financial education and policy efforts with the goal of improving overall quality of life.

## Figures and Tables

**Table 1:** Summary statistics (by gender and DFL).

	Full Sample		By Gender		By DFL	
	Mean	SD	Male	Female	DFL ≤ Median	DFL > Median
Life satisfaction	60.92	19.17	59.83	62.06 *	58.61	63.22 *
Digital financial literacy ^a^	22.65	2.88	22.85	22.43 *	20.48	24.82 *
Financial knowledge ^a^	5.44	1.45	5.58	5.30 *	4.64	6.24 *
Digital literacy ^a^	4.62	0.56	4.62	4.61	4.31	4.92 *
Digital financial service awareness ^a^	4.43	0.62	4.45	4.42	4.06	4.81 *
Practical know-how of digital financial services ^a^	4.40	0.61	4.40	4.40	4.04	4.76 *
Self-protection against digital financial fraud ^a^	3.75	0.65	3.81	3.70 *	3.43	4.08 *
Age	42.63	9.67	42.59	42.67	43.91	41.34 *
Male	(51.2)				(45.7)	(56.8)
Female	(48.8)				(54.3)	(43.2)
High school graduate	(18.5)		(16.7)	(20.4)	(24.3)	(12.8)
Vocational college graduate	(16.9)		(15.2)	(18.7)	(19.7)	(14.1)
4-year college graduate	(53.1)		(53.9)	(52.2)	(48.8)	(57.4)
Postgraduate degree	(11.5)		(14.1)	(8.8)	(7.3)	(15.7)
Single	(33.8)		(38.0)	(29.4)	(32.3)	(35.3)
Married	(59.3)		(56.7)	(61.9)	(59.9)	(58.6)
Separated/divorced/widowed	(6.9)		(5.3)	(8.6)	(7.8)	(6.1)
Household size	2.93	1.22	2.95	2.92	2.93	2.94
Self-rated health: very poor, poor	(11.5)		(12.9)	(9.9)	(14.1)	(8.8)
Self-rated health: average or better	(88.5)		(87.1)	(90.1)	(85.9)	(91.2)
Household income (KRW 10k)	10,418	39,973	9599	11,276	9428	11,408
Financial assets (KRW 10k)	13,219	47,074	12,552	13,920	11,418	15,023
Poverty status	(1.7)		(1.7)	(1.8)	(2.6)	(0.9)
Own home	(60.1)		(60.1)	(60.0)	(60.4)	(59.7)
Long-term rental	(17.9)		(17.8)	(18.0)	(17.6)	(18.2)
Short-term rental and others	(22.0)		(22.1)	(22.0)	(22.0)	(22.1)
N	1615		827	788	808	807

Notes: DFL, digital financial literacy; SD, standard deviation; N, number of observations. Percentage figures in parentheses. ^a^ Raw scores before normalization. * Denotes significant difference between group at the 5% level, according to two-sample *t*-test for continuous variables.

**Table 2:** Correlation matrix.

	Life Satisfaction	Digital Financial Literacy	Financial Knowledge	Digital Literacy	Awareness of DFS	Practical Know-How of DFS	Self-Protection
Life satisfaction	1.000						
Digital financial literacy	0.149	1.000					
Financial knowledge	0.083	0.727	1.000				
Digital literacy	0.115	0.750	0.280	1.000			
Awareness of DFS	0.132	0.817	0.333	0.735	1.000		
Practical know-how of DFS	0.125	0.811	0.282	0.749	0.839	1.000	
Self-protection	0.138	0.636	0.176	0.448	0.519	0.594	1.000

Notes: DFS, digital financial services.

**Table 3:** Associations between digital financial literacy and life satisfaction.

	(1)	(2)	(3)
Digital financial literacy	2.863 ***	1.946 ***	1.752 ***
	(0.472)	(0.464)	(0.447)
Age		−0.053	−0.038
		(0.055)	(0.053)
Female		2.967 ***	2.182 **
		(0.905)	(0.853)
Vocational college graduate		1.593	0.461
		(1.512)	(1.441)
4-year college graduate		7.724 ***	6.028 ***
		(1.249)	(1.201)
Postgraduate degree		14.196 ***	11.235 ***
		(1.727)	(1.651)
Married		8.492 ***	6.734 ***
		(1.212)	(1.172)
Separated, divorced, widowed		−1.340	−0.726
		(1.996)	(1.894)
Household size		0.051	−0.332
		(0.408)	(0.394)
Self-rated health: average or better			18.110 ***
			(1.350)
Household income			0.003 **
			(0.001)
Financial assets			0.001
			(0.001)
Poverty status			4.058
			(3.296)
Jeonse (long-term rental)			−0.128
			(1.175)
Monthly rent/other			−4.694 ***
			(1.133)
Adj. R^2^	0.02	0.13	0.23

Notes: Standard errors in parentheses. Regression in column (3) controls for province-fixed effects. *** *p* < 0.01; ** *p* < 0.05; * *p* < 0.10.

**Table 4:** Associations between digital financial literacy subdimensions and life satisfaction.

	(1)	(2)	(3)	(4)	(5)	(6)	(7)
Financial knowledge	0.850 *					0.452	
	(0.438)					(0.460)	
Digital literacy		1.606 ***				1.168 *	
		(0.443)				(0.675)	
Awareness of DFS			1.313 ***			−0.200	
			(0.445)			(0.831)	
Practical know-how of				1.432 ***		−0.145	
DFS				(0.445)		(0.888)	
Self-protection against					1.687 ***	1.318 **	
digital financial fraud					(0.435)	(0.530)	
Digital financial literacy							1.752 ***
							(0.447)
Adj. R^2^	0.22	0.23	0.22	0.22	0.23	0.23	0.23

Notes: DFS, digital financial services. Standard errors in parentheses. All regressions control for age, gender, education, marital status, household size, self-rated health, household income, financial assets, poverty status, housing type, and province-fixed effects. *** *p* < 0.01; ** *p* < 0.05; * *p* < 0.10.

## Data Availability

The original contributions presented in this study are included in the article. Further inquiries can be directed to the corresponding author.

## Appendix A

**Table A1:** Measurement items for digital financial literacy scale.

	Mean	SD
Financial knowledge (α = 0.55)		
When spending 1 million won on a specific product, how does the amount of goods you can buy now compare to what you can buy in a year? (Assuming an inflation rate of 3%)	0.85	0.36
You lent a friend 100,000 won, and they repaid 100,000 won the next day. In this case, how much interest did your friend pay on the loan?	0.70	0.46
If you deposit 1 million won into a 1-year fixed deposit account with a guaranteed annual interest rate of 2%, without fees and taxes, and with no additional deposits or withdrawals, how much money will be in the account after 1 year?	0.76	0.43
If you deposit 1 million won into a 1-year fixed deposit account with a guaranteed annual interest rate of 2%, without fees and taxes, and with no additional deposits or withdrawals, how much money will be in the account after 5 years?	0.52	0.50
Investments with higher returns are likely to involve greater risks.	0.97	0.18
High inflation means that the cost of living is increasing rapidly.	0.89	0.31
Generally, you can reduce investment risk in the stock market by buying a variety of stocks.	0.78	0.42
Digital literacy (α = 0.96)	4.62	0.56
I know how to turn digital devices on and off	4.67	0.60
I know how to charge digital devices	4.69	0.58
I know how to authenticate myself on digital devices using text messages, KakaoTalk, or biometric authentication	4.62	0.65
I know how to authenticate myself on digital devices using fingerprint, facial, or iris recognition	4.56	0.73
I know how to connect digital devices to the internet using Wi-Fi	4.67	0.61
I know how to install programs/applications on computers or smartphones	4.65	0.65
I know how to update smartphone applications	4.66	0.62
I know how to sign up for websites or smartphone apps and manage accounts	4.66	0.62
I know how to log in to internet websites or smartphone applications	4.66	0.63
I know how to adjust and manage the privacy settings of my member account	4.31	0.86
Digital financial service awareness (α = 0.90)	4.43	0.62
I know that I can do banking tasks through internet banking or smartphone apps	4.73	0.55
I know that I can do investment tasks through securities company websites or smartphone apps	4.37	0.91
I know that loans are possible online or through apps without visiting in person	4.44	0.81
I know that it’s possible to pay fees using online payment or simple payment systems	4.65	0.63
I know the purpose and usage of online/mobile banking	4.53	0.68
I know the purpose and usage of online/mobile stock trading	4.20	0.99
I know the purpose and usage of online/mobile loan services	4.13	0.98
I know the purpose and usage of online/mobile simple payment systems	4.40	0.76
Practical know-how of digital financial services (α = 0.90)	4.40	0.61
I can sign up for digital financial services through smartphone apps	4.56	0.68
I can authenticate myself for digital financial services using smartphone simple authentication	4.59	0.65
I know how to handle websites or apps related to digital financial services	4.37	0.79
I can perform basic financial transactions through digital financial services	4.47	0.76
I can use smartphone simple payment functions	4.62	0.65
I know how to cancel a transaction if an error occurs in digital financial services	3.90	0.97
I can contact customer service to resolve issues if an error occurs in digital financial services	4.29	0.79
Self-protection against digital financial fraud (α = 0.84)	3.75	0.65
I know how to avoid unnecessary fees when conducting online/mobile financial transactions	3.80	0.95
I can identify bait products in online/mobile financial transactions	3.70	0.92
I pay attention to password management for logging into digital financial services	3.97	0.90
I regularly change passwords for logging into digital financial services	3.21	1.05
I know how to respond if a password is leaked	3.44	1.03
I don’t click on URL links sent from unknown financial institutions	4.42	0.75
I regularly check with malware detection programs	3.57	1.08
I have the ability to identify and avoid voice phishing	3.92	0.84

Notes: SD, standard deviation.
